# Forensic Discrimination of Differentially Sourced Animal Blood Using a Bottom-Up Proteomics Based MALDI MS Approach

**DOI:** 10.3390/molecules27072039

**Published:** 2022-03-22

**Authors:** Katie Kennedy, Laura Cole, Matthias Witt, Mark Sealey, Simona Francese

**Affiliations:** 1 Biomolecular Research Centre, Sheffield Hallam University, Sheffield S1 1WB, UK; kk6068@exchange.shu.ac.uk (K.K.); l.cole@shu.ac.uk (L.C.); 2 Bruker Daltonics GmbH & Co. KG, 28359 Bremen, Germany; matthias.witt@bruker.com; 3 Defence Science and Technology Laboratories (DSTL), Porton Down SP4 0JQ, UK; msealey@mail.dstl.gov.uk

**Keywords:** blood, MALDI, forensic

## Abstract

Recently published work has reported the development and application of a bottom-up proteomic approach to distinguish between human and animal blood (down to animal species level), by rapid screening using Matrix Assisted Laser Desorption Ionisation Mass Spectrometry (MALDI MS). In that study, it was additionally observed that intravenous animal blood exhibits different spectral profiles from blood collected within the animal chest cavity as well as from the diluted blood collected within packets of meat. In this follow-up study we explored the resulting hypothesis that, depending on how blood is shed or collected, protein biomarker profiles vary to the extent of systematically permitting a distinction between possible sources of blood (for example, flesh wound versus packaged meat). This intelligence may be important in reconstructing the dynamics of the crime. The combination of statistical analysis and tandem mass spectrometry has yielded additional animal blood markers as well as confirming the ability to correctly determine the animal species from which blood derived, regardless of the retailer selling it (amongst the five investigated). These data confirm the initial hypothesis and demonstrate the opportunity for the proteomics-MALDI combined approach to provide additional intelligence to the investigation of violent crimes when examining blood evidence.

## 1. Introduction

The specific detection of blood and other biofluids at crime scenes is of vital importance to correctly understand the circumstances and the nature of a violent crime. The currently deployed presumptive tests, such as BlueStar Forensic (Luminol), Kastle-Meyer test and some acid dyes for blood stain/mark enhancement can, on occasion, give rise to false positives [1], which may result in a case being dismissed due to the validity of these tests’ results being brought into question [2]. Additionally, without the use of a serological test, blood provenance, which is important additional intelligence, cannot be determined. Whilst liquid chromatography tandem mass spectrometry (LC MS/MS) remains the gold standard method to provide comprehensive proteomic information, over recent years, Matrix Assisted Laser Desorption Ionisation Mass Spectrometry (MALDI MS) has been explored as (i) a rapid confirmatory test for the presence of blood [3,4,5,6], (ii) as a source of additional intelligence around blood provenance [4,7,8,9], (iii) to aid suspect or victim identification [10] and (iv) to visualise the presence of blood in fingermarks [2,3,10,11] compatibly with the prior application of blood enhancement techniques and, recently, with the subsequent application of DNA typing [2].

With regards to establishing blood provenance, Bradshaw et al. [3] and Patel et al. [4] first showed the possibility of determining blood provenance in blood marks discriminating between equine, bovine and human blood using a direct MALDI MS Profiling/Imaging (MSP/MSI) approach or combined with bottom-up proteomics. Kamanna et al. [9] successfully showed that detection and visualisation of both human and animal blood (from a selection of native Australian animals) in fingermarks was possible using intact and bottom-up proteomic approaches with direct MALDI MSI analysis of alpha (α) and beta (β) Haemoglobin (Hb) chains and their derived peptides. A study by Lecrenier et al. [12], in addition to detecting several blood specific proteins including Hb (α and β chains), Serotransferrin, and Apolipoprotein A1, also found specific bovine blood markers.

**Figure 1:**
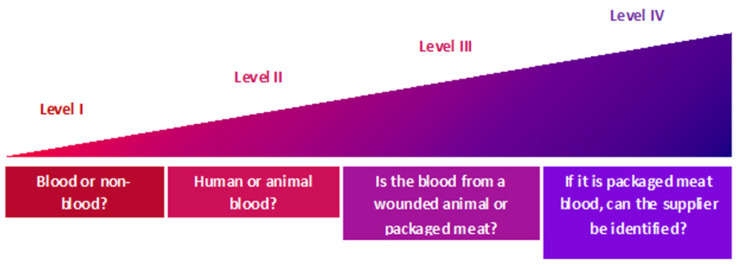
Schematics of the possible levels of identification for the detection of human and animal blood using the bottom-up proteomic MALDI MS based approach. Revised from Kennedy et al. [7].

In a pre-validation study, Kennedy et al. [7] established the feasibility of MALDI MS-based methods to act as a confirmatory test, distinguishing between human and animal blood, down to species level (bovine, chicken and porcine), also confirming some of the markers detected by Lecrenier et al. [12]. In this study [7], animal blood was sourced from the (i) jugular vein, which in the present paper will be referred to as “intravenous”, and from (ii) packaged meat. An interesting observation was made in that for each of the selected animal species, the MALDI mass spectrum of the digested blood differed according to where the blood was sourced from, both in signal intensity and in terms of peptide profiles. Ultimately, mass spectral differences appeared to relate to the location from where blood was collected. For example, the mass spectral profile of intravenous blood was not superimposable to that of blood collected from the chest cavity of the animal. This occurrence led to the hypothesis that, in addition to the ability to detect blood and to differentiate between human and animal blood (down to animal species), it may also be possible to provide intelligence with respect to how the animal blood was generated at the crime scene through a specific panel of markers. The body of knowledge derived on animal blood detection and source discrimination from the Kennedy et al. work [7] has been adapted and integrated in a new “identification levels” schematic (Figure 1) hypothesising the potential to gain further insight surrounding the circumstances of animal bloodshed.

The ability to ascertain whether animal blood derived from a wounded animal at the scene or from someone handling raw meat may be important to some investigations. In the latter case, as an example, this information would have been helpful in the UK murder case of Susan May (Regina vs. Susan May), to corroborate or disprove her statement, claiming that if blood was indeed present in her fingerprint on the bedroom’s wall, it would have been of animal origin, and specifically from raw packaged meat, due to her handling it to prepare lunch for her aunt.

The 1996 US case, State v. Leuluaialii, Nos. 96-C-08256-9, 97-C-01391-3 (Wash., King County Super. Ct. 16 September 1998) is an example where blood from a wounded animal was crucial evidence. Within that double homicide case, the blood of the dog shot dead together with its owners was recovered from the clothing of the offenders [13]. Whilst in that case, canine DNA could be recovered from the bloodstain placing the suspects at the scene (and aiding conviction), in instances where DNA typing is not possible, the determination of the presence of blood, its animal origin and down to animal species would provide the same intelligence.

In the present adaptation of the Kennedy et al. work [7], identification level III describes the discrimination of blood provenance (within each animal species), between intravenous and packaged meat. Whilst the former scenario refers in our work to the case of wounded animals, the latter scenario refers to an individual handling packaged raw meat and, thereby, contaminating their fingertips with what is most likely a mixture of diluted blood, preservatives and proteins/other biomolecules released directly from the flesh into the blood as a result of severe muscle damage. Level III is the predominant focus of the present follow-up study, but the discovery of additional biomarkers of bovine, chicken and porcine blood was also pursued to increase robustness and reliability of the method.

Identification level IV delves further into species and source discrimination for blood in packaged meat. The robustness of the method was confirmed by establishing that blood originating from packaged meat could still be correctly assigned to the corresponding animal species, regardless of the food retailer from which it was purchased. A subsequent hypothesis that was addressed concerned the assessment of whether blood from packaged meat could be traced back to a specific supermarket (given that the food processing and supply chains could be different for different supermarkets). This type of intelligence may be important to corroborate/disprove the defendant’s statement or for gathering additional evidence (such as shopping receipts, reviewing CCTV recordings) by tracing the provenance of the blood in the packaged meat down to a retailer. However, this type of intelligence could only be partially obtained in the present study and indicated that there may only be a few differences in the chain supplies/food processing for the different UK supermarkets investigated.

## 2. Results and Discussion

### 2.1. ID Level III: Differentiation between Blood Simulating a Wounded Animal (Intravenous) Versus Blood in Packaged Meat

In the study by Kennedy et al. [7], a method for in solution proteolysis and MALDI MS profiling of chicken, porcine and bovine blood was developed and pre-validated yielding blood biomarkers for each species. However, the study focused on the identification of protein markers in blind samples originating from blood present in butchered meat (which was collected from the chest cavity of the animal), rather than blood sourced directly from the jugular vein (intravenous). The spectral profiles acquired from the chest cavity of the butchered animals were not superimposable with the spectra from the blood found in the packaged meat. These observations suggested that it may be possible to establish the “source” or “type” of blood encountered at the scene. The ability to distinguish between intravenous and packaged meat blood (and other types) would facilitate reconstruction of the crime scene dynamics and the events leading to the presence of animal blood, where involved. For this reason, in the present study, intravenous blood was further investigated using statistical analysis to assess MALDI spectra profiles and candidate markers were searched for in the *m*/*z* range 1100–2000 (to minimise matrix/trypsin peaks as well as variability of ion signals above *m*/*z* 2000).

#### 2.1.1. Animal Species Determination from Intravenous Blood Simulating a Wounded Animal (Collected from the Jugular Vein)

**Figure 2:**
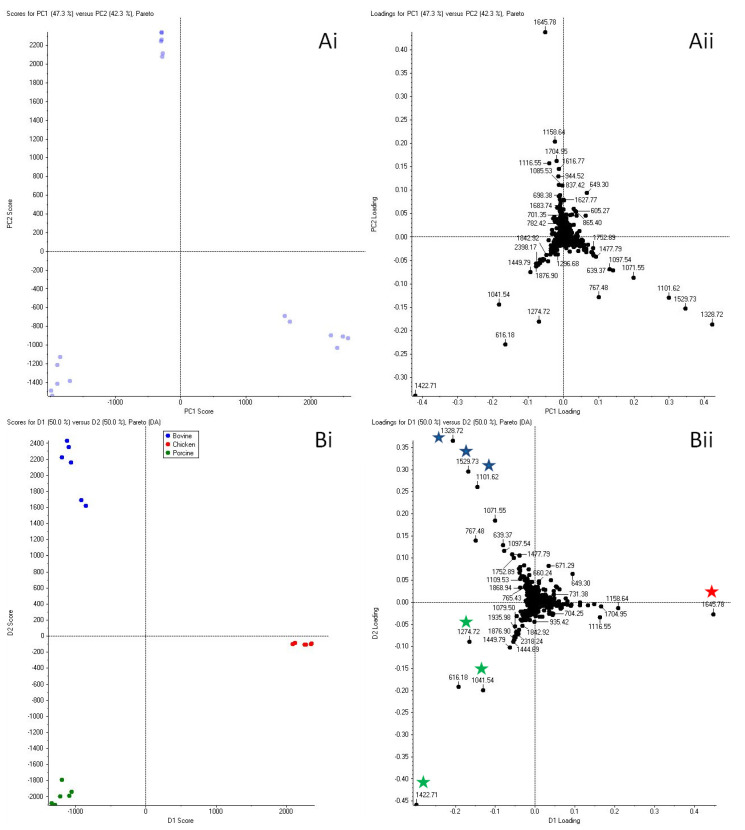
Principal component analysis of bovine, chicken and porcine blood collected from the jugular vein of the animal (“intravenous”). Six replicate spectra from each animal blood in solution proteolytic digests were used for PCA. Clear and distinct grouping can be observed with all three species in both the unsupervised (**Ai**,**Aii**) and supervised PCA-DA analyses (**Bi**,**Bii**). In (**Ai**), the purple dots on the scores plot are to show the variance and spatial relationship of the group replicates from an ‘un-supervised’, non-bias way hence no color has been pre-assigned to them. The unsupervised score plot in (**Ai**) clearly aligns with that of the supervised PCA-DA validating the rational for labelling. The star symbol in (**Bii**) indicates the ion signals subjected to MALDI MS/MS analysis. The colors in (**Bii**) refer to the animal species as color referenced in (**Bi**).

Principal Component Analysis (PCA) and Principal Component Analysis–Discriminant Analysis (PCA-DA) were performed to objectively demonstrate the difference in blood biomarker composition between intravenous bovine, chicken, and porcine blood. The blood marker panels observed seemed to vary, with the most intense proteotypic signal(s) for each species (within the animal species and proteins being investigated) being present alongside a group of additional peptides, amongst which only some were shared across multiple species. It is this combination of markers, as opposed to the individual theoretical peptides from the in-silico digestion, that is truly discriminatory and acts as a ‘species identifying’ panel of ion signals. The ion signals mostly responsible for the different clusters resulting from PCA of the mass spectra of intravenous animal blood were subjected to MALDI MS/MS analyses and identifications are discussed in the following Sections. These analyses permitted the expansion of the panel of identified markers indicative of bovine, porcine and chicken intravenous blood (Supplementary Table S1 and Figure S1). As shown in Figure 2, the signal at nominal *m*/*z* 1329 was the most discriminatory signal for bovine blood in the loading plot, although the ion signals at nominal *m*/*z* 1102 and 1530 were also included in the biomarker panel for bovine intravenous blood.

The ion signal at nominal *m*/*z* 1646 was by far the most discriminatory for the intravenous chicken blood. In porcine blood, the signal at nominal *m*/*z* 1423 was most responsible for the clustering of porcine blood away from the other animal blood, although the ion signals at nominal *m*/*z* 1042 and 1275 were also included in the porcine intravenous blood biomarker panel.

##### ID Level III: Intravenous Bovine Blood Marker Identification

The only blood peptide signatures detected in the digested intravenous bovine blood were putatively assigned to Hb and characterised by a mass accuracy between −3.7 and 4.7 ppm (Table 1).

Several of these signals were proteotypic to bovine blood. However, despite several signals observed in the intravenous bovine blood being proteotypic (within the species and proteins investigated), very many were not responsible for the clustering of the bovine blood in the PCA; therefore, only the ion signals indicated by the PCA as discriminatory, at nominal *m*/*z* 1102, 1329, 1530 MS/MS were subjected to MS/MS analysis (Supplementary Table S1). The signal at *m*/*z* 1328.727 was the most intense and putatively assigned to bovine βHb. MALDI MS/MS analysis and an automatic MASCOT MS/MS search confirmed its identity as foetal bovine βHb (VKVDEVGGEALGR) despite the putative identification indicating adult bovine βHb (Supplementary Table S1 and Figure S1A). Indeed, this sequence is found in both bovine foetal βHb and adult bovine βHb and is proteotypic to bovine blood. The signal detected at *m*/*z* 1529.745 and corresponding to αHb (VGGHAAEYGAEALER) was also indicated in the PCA loading plot as one of the signals responsible for bovine intravenous blood discrimination. However, this peptide is also found in the human αHb sequence; therefore, only when the signals at nominal *m*/*z* 1329, 1102 and 1530 are present together can they be utilised as the (combined) unique protein signature for ‘intravenous’ bovine blood. Notwithstanding, due to the low intensity of the signal at nominal *m*/*z* 1102, MS/MS analysis was not possible, and the identity of this *m*/*z* signal remains unconfirmed.

##### ID Level III: Intravenous Porcine Blood Marker Identification

As for the intravenous bovine blood, several porcine blood proteotypic signals were detected and putatively assigned within a mass accuracy between −3.8 and 8.3 ppm (Table 2).

##### ID Level III: Intravenous Chicken Blood Marker Identification

Every putative peptide match in the MALDI MS spectrum of intravenous chicken blood was assigned to αHb or βHb (Table 3); these peptides were proteotypic to chicken blood (within the animal species and blood specific proteins being investigated) and having a mass accuracy ranging between −1.8 and 14.6 ppm.

The ion at *m*/*z* at 1645.791 is a suitable positive marker for intravenous chicken blood due to (i) causing the most clustering of chicken blood in the PCA plot, (ii) its high intensity and (iii) it being proteotypic to chicken blood (within the proteins and animal species being investigated). Its assignment to αHb (IAGHAEEYGAETLER) was confirmed through MALDI MS/MS analysis (Supplementary Table S1 and Figure S1C).

#### 2.1.2. ID Level III: Statistical Analysis Discrimination between Intravenous Blood (Mimicking a Wounded Animal) Versus Blood in Packaged Raw Meat

Once discrimination between all animal species could be established through the MALDI MS profile of their intravenous blood it became important to verify the hypothesis made by Kennedy et al. [7] that it may be possible to distinguish between intravenous blood and blood found in packaged meat.

**Figure 3:**
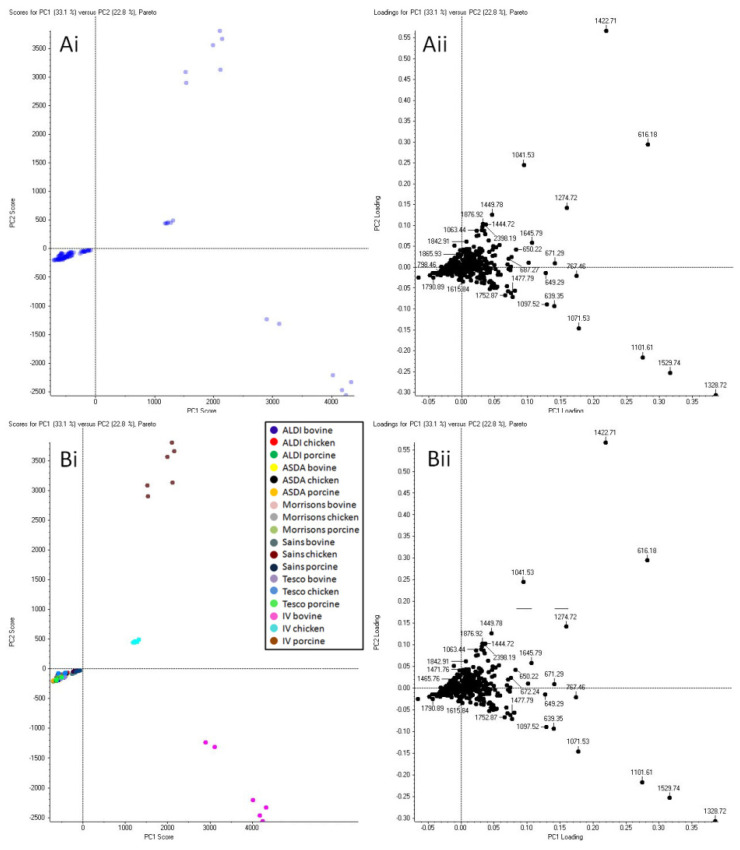
PCA of bovine, chicken and porcine blood collected from the jugular vein of the animal versus bovine, chicken and porcine blood collected from packaged raw meat for each species (from the same five supermarkets). Six replicate spectra from each in solution animal blood proteolysis were used for the statistical analysis. (**Ai**,**Bi**) show the unsupervised and supervised PCA score plots whereas (**Aii**,**Bii**) the unsupervised and supervised loading plots, respectively, where (**Bi**,**Bii**) are the outputs of the PCA-DA.

Figure 3 shows both the unsupervised and supervised PCA of intravenous and packaged meat blood for chicken, porcine and bovine animal species, with packaged meat sourced from five different supermarkets (Aldi, Asda, Morrison’s, Tesco and Sainsbury’s). PCA shows a clear separation between intravenous blood and blood sourced from packaged meat. The clustering does not change significantly between the unsupervised and supervised analyses, thus further verifying the hypothesis made.

### 2.2. ID Level III: Species Determination from Blood in Packaged Meat

**Figure 4:**
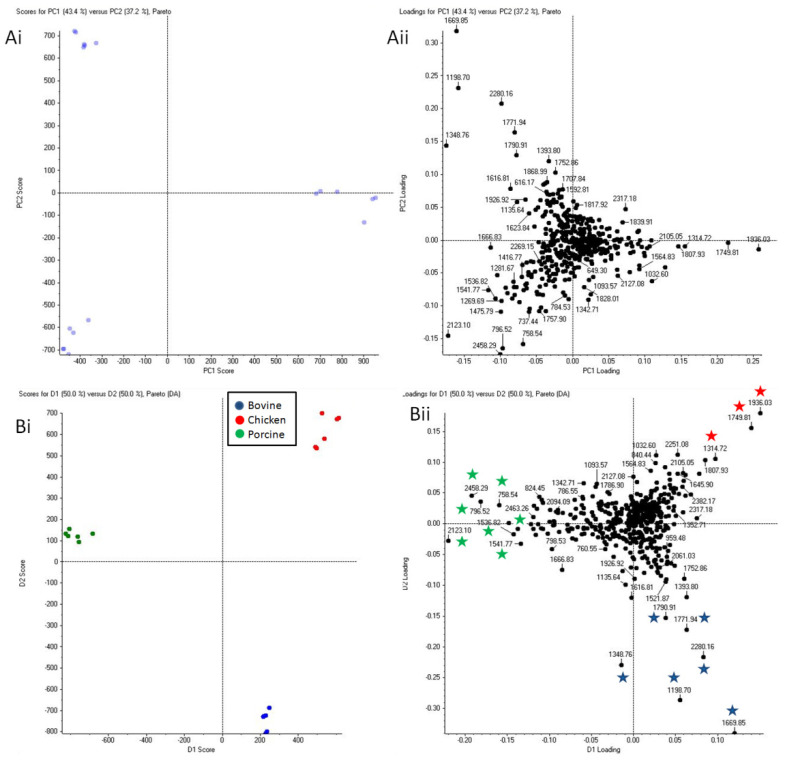
PCA of bovine, chicken and porcine blood collected from packaged raw meat (from just one supermarket namely Aldi). Six replicate spectra from each in solution digest were used for PCA. (**Ai**,**Bi**) show the unsupervised and supervised PCA score plots whereas (**Aii**,**Bii**) the unsupervised and supervised loading plots, respectively. The color stars in (**Bii**) refer to the animal species as color referenced in (**Bi**). The star symbol indicates the *m*/*z* ion signals selected for MALDI MS/MS analysis.

The PCA shown in Figure 3 was “deconstructed” to delve deeper into the intelligence that could possibly be gained in relation to the potential of narrowing down the origin of the packaged meat blood to a retailer. It was preliminarily assessed whether animal species clustering could be observed for blood in packaged meat purchased from only one supermarket. Figure 4 shows the PCA plots for the MALDI MS profiles of blood in packaged chicken, bovine, and porcine meat from one randomly chosen supermarket (Aldi). Clear clustering is visible in both PCA plots indicating that animal species differentiation for packaged meat is also possible (within the system investigated). The ion signals most responsible for animal species clustering and identified in the loading plots (starred in Figure 4) were putatively identified and then subjected to confirmatory MALDI MS/MS analysis (Table 4).

#### 2.2.1. ID Level III: Bovine Packaged Blood Marker Identification

For bovine packaged blood, the signals at nominal *m*/*z* 1199, 1349 and 1670 were the most discriminatory and were putatively identified as Actin, Carbonic Anhydrase 3 and Myoglobin respectively and were also confirmed by MS/MS analysis (Table S2). The ion signals at nominal *m*/*z* 1791 and 2280 were putatively identified as Actin and Myoglobin respectively; though the signal at nominal *m*/*z* 1772 could not be putatively identified, it was confirmed to belong to Carbonic Anhydrase 3 through MALDI MS/MS analysis. The detection of both Actin and Myoglobin for blood in packaged meat is expected as they are found in muscle tissue and skeletal muscle tissue respectively. Carbonic Anhydrase 3 is a metalloenzyme present in tissues where a high rate of oxygen consumption occurs [14]. This protein catalyses the reversible hydration of carbon dioxide to bicarbonate and proton and has been reported to play an antioxidant role in the presence of oxidative stress/damage [14]. In Kennedy et al. [7], ion signals at nominal *m*/*z* 1593 and 1670 belonging to Myoglobin were indicated as positive markers for bovine blood collected from the chest cavity of the animal.

#### 2.2.2. ID Level III: Porcine Packaged Blood Marker Identification

For the packaged porcine blood, the ion signals at nominal *m*/*z* 758, 796, 1537, 1542, 2123, 2458 and 2463 were observed as the most discriminatory and selected for putative and MALDI MS/MS identification. The ions at *m*/*z* 758.576 and 796.533 were clearly identified as phosphatidylcholines through accurate mass and the presence of a phosphocholine headgroup ion fragment at *m*/*z* 184.074 (data not shown). Despite being a relatively high intensity and densely ion populated MS/MS spectrum, it was not possible to identify the ion at *m*/*z* 1536.823, likely due, from inspection of the spectrum, to more than one parent ion being selected for fragmentation. The ion at *m*/*z* 1541.766 was putatively identified as Beta-Enolase (LAQSNGWGVMVSHR) and was confirmed through MALDI MS/MS analysis with a mass accuracy of fragments between −13.6 and 2.1 ppm. Despite no putative identification of the ion at *m*/*z* 2463.248, upon MALDI MS/MS analysis, an automatic MASCOT MS/MS search identified this signal as the sequence AAVPSGASTGIYEALELRDGDKSR, also belonging to Beta-Enolase, with a mass accuracy across the ion fragments ranging between −10.9 and 10.7 ppm. This protein is found in striated muscle and is involved in a sub-pathway of glycolysis. The ion signal at *m*/*z* 2123.125 has been identified by MALDI MS/MS as the sequence IGEHTPSSLAIMENANVLAR belonging to Fructose-Bisphosphate Aldolase; another protein also involved in a sub-pathway of glycolysis. The ion signal at *m*/*z* 2458.314 could not be identified putatively or through MS/MS analysis.

#### 2.2.3. ID Level III: Chicken Packaged Blood Marker Identification

**Figure 5:**
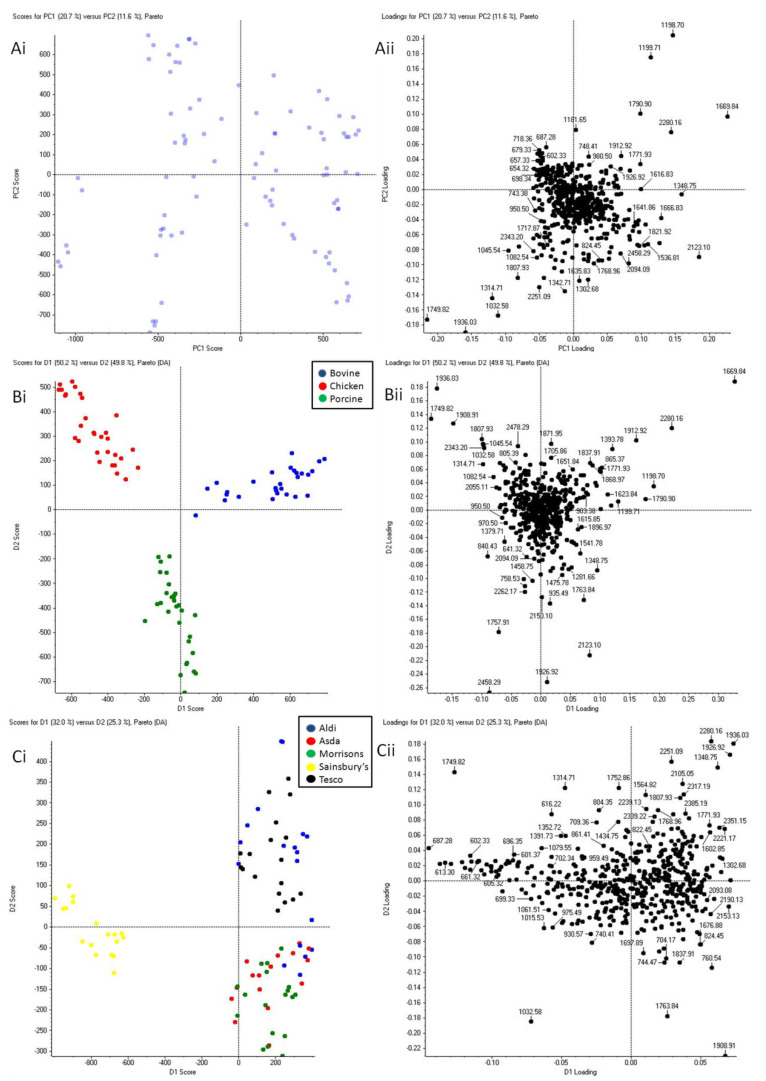
Principal component analysis of bovine, chicken and porcine blood collected from raw packaged meat (from five supermarkets in total: Asda, Aldi, Morrison’s, Tesco, and Sainsbury’s). Six replicate spectra from each in solution packaged meat animal blood proteolysis were used for the statistical analysis. Panels (**Ai**,**Aii**) are the unsupervised PCA plots; Panels (**Bi**,**Bii**) are the supervised PCA plots with grouping based on meat types; Panels (**Ci**,**Cii**) are the supervised PCA plots with grouping based on supermarkets.

For the packaged chicken blood, the ion signals selected from the PCA (Figure 4) as the most discriminatory were at nominal *m*/*z* 1315, 1750 and 1936. Of these ion signals, only that at *m*/*z* 1749.799 could be identified and assigned to the Glyceraldehyde-3-Phospate Dehydrogenase (GAPDH) sequence LVSWYDNEFGYSNR already detected in the blood from the chest cavity of the animal by Kennedy et al. [7]. Where ions could not be identified by MS/MS analysis, this was due to a combination of (i) the known challenge in performing tandem MS on singly charged ions using MALDI (especially for higher *m*/*z* ions), as opposed to using LC MS/MS analysis, and (ii) the simultaneous selection of multiple precursor ions yielding mixed MS/MS spectra. Subsequently, we sought to ascertain whether the blood from different animal species could be correctly discriminated regardless of the retailer source. For this investigation, five major UK retailer sources were used. From the PCA plots reported in Figure 5, it can be observed that whilst the unsupervised analysis shows no clustering (Figure 5(Ai,Aii)), the supervised analysis shows clear separation between all the chicken, porcine and bovine blood regardless of the source (retailer) of packaged meat (Figure 5(Bi,Bii)). Figure 5(Ci,Cii) show supervised PCA performed with grouping based on supermarkets to ascertain whether the blood from all packaged meat, across all animal species, could cluster according to the supermarkets of provenance. In this case, there was no clustering except for the Sainsbury’s packaged meat. This observation may be beneficial intelligence in a forensic scenario as it could help identify a specific supermarket from which the animal blood could have originated and help corroborate the suspect’s testimony or investigative evidence.

### 2.3. ID Level IV: Assessment of the Potential to Identify the Retailer for the Animal Blood in Packaged Meat

**Figure 6:**
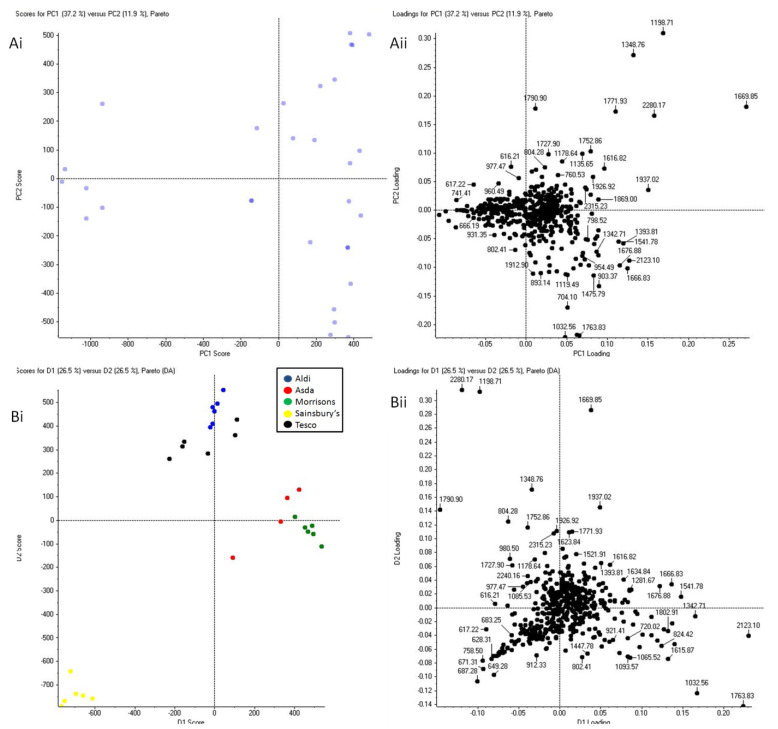
Principal component analysis of bovine blood collected from raw packaged meat (from five supermarkets in total: Asda, Aldi, Morrison’s, Tesco, and Sainsbury’s). Six replicate spectra from each in solution packaged bovine blood proteolysis were used for the statistical analysis. Panels (**Ai**,**Aii**) are the unsupervised PCA plots; Panels (**Bi**,**Bii**) are the supervised PCA-DA plots with blood provenance grouping based on supermarket of origin.

In a final investigation, to provide further assistive intelligence to violent crime investigations it was sought to ascertain whether it was possible to determine the provenance in terms of supermarket retailer from which the packaged meat blood originated. Figure 6 shows both unsupervised and supervised PCA of blood spectral profiles in packaged meat from five supermarkets, but only for bovine blood as an example.

PCA plots of blood collected from packaged meat purchased from five different supermarkets are shown in Figure 6(Bi,Bii) groups. Some level of clustering can be observed for the blood from Morrison’s packaged bovine meat and for the blood from Aldi’s packaged bovine meat. However, whilst Morrison’s and Aldi’s can be distinguished from each other, they could be easily mistaken for Asda’s and Tesco’s, respectively. Bovine blood from Sainsbury’s packaged meat remains, in this analysis too, clearly distinguishable from the bovine blood deriving from other retailers in their corresponding packaged bovine meat. From these observations, it can be speculated that Sainsbury’s may have a different meat supplier and/or rely on different food processing. The same speculation can be made for Morrison’s and Aldi, although, from these analyses, they seem to share supplier/food processing with Asda and Tesco respectively.

## 3. Materials and Methods

### 3.1. Materials

Trifluoracetic acid (TFA), α-cyano-4-hydroxycinnamic acid (α-CHCA) was purchased from Sigma Aldrich (Poole, UK). Acetonitrile (CAN) was purchased from Fisher Scientific (Loughborough, UK). Sequencing grade modified lyophilized Trypsin was purchased from Promega in 20 µg vials (Southampton, UK). Sigma dry tubed swabs were sourced from Medical Wire (MWE) (Wiltshire, UK) and Rapigest was obtained in 1 mg vials from Waters (Wilmslow, UK). Polylysine slides were obtained from Thermo Scientific (Altrincham, UK). Intravenous blood samples (bovine, chicken and porcine, 1 mL defibrinated and 1 mL with EDTA for each animal) were purchased from TCS Biosciences (Buckingham, UK). Packets of chicken, rump steak and pork chops were sourced from Sainsbury’s, ASDA, Tesco, Morrison’s, and ALDI (Sheffield, UK). The white meat was sourced from the breast of the chicken, the rump steak was taken from the hindquarter and muscle above the hipbone, and the pork chop was from the loin of the animal, which is located from the hip to the shoulder of the pig. All meat acquired for this study was fresh and in date and kept refrigerated with all analyses performed on the day of purchase.

### 3.2. Methods

#### Enzymatic Digestion of Blood

Rapigest (0.1% *v*/*v* in 50 mM ammonium bicarbonate solution) was added to trypsin to reconstitute it in a 20 µg/mL solution. For the animal blood from the jugular vein of each animal, 10 μL of blood were spotted onto a polylysine slide and then swabbed with 70:30 ACN: H_2_O; for packaged meat, the diluted blood at the bottom of each packet was collected using a swab. The swab head was removed using scissors and transferred into an eppendorf where 1 mL of 70:30 ACN: H_2_O solution was added prior to sonication for 10 min. Ten µL of the 1 mL extract were added to 40 µL of 40 mM ammonium bicarbonate and to 9 µL of trypsin solution at a concentration of 20 µg/mL and Rapigest (0.1% *v*/*v*). The sample was then incubated for 1 h at 37 °C and the proteolytic digestion was stopped with the addition of 2 µL of 5% TFA.

### 3.3. Instrumental Conditions

#### 3.3.1. MALDI MS and MS/MS

All MALDI MS spectrometric analyses were carried out using the Waters MALDI-QTOF Synapt G2 HDMS instrument (Waters Corporation, Manchester, UK). Data acquisition was performed within the *m*/*z* range 600–2500 Th in positive sensitivity mode. The Nd: YAG laser repetition rate was set to 1 kHz for all analyses.

MALDI MS/MS spectra for intravenous animal blood markers were obtained on the MALDI-QTOF Synapt G2 HDMS instrument and on a timsTOF flex system (Bruker Daltonik, Bremen, Germany) for packaged meat blood markers.

For the Synapt MALDI instrument, argon was used as the collision gas. The trap collision energy was set between 30 eV and 90 eV depending upon the precursor *m*/*z* (higher collision energy for higher *m*/*z*). The laser power and low mass resolution were set at 250 and 14.6, respectively. A 0.5 µL spot of saturated phosphorus red solution in ACN was used as an internal calibrant in the *m*/*z* range 600–2500 Th prior to analysis.

For the timsTOF flex MALDI instrument, data acquisition was performed in the *m*/*z* range 100–3000 Th in positive ion mode. The instrument was equipped with a smartbeam 3D laser operated at 10 kHz, with the laser focus set for MS/MS experiments. The laser power was set between 35–45% and 500 to 1000 laser shots per scan were acquired (10–20 scans were added for generation of final spectrum). The collision energy was set between 40 eV and 125 eV again depending on the *m*/*z* of the precursor.

#### 3.3.2. Matrix and Application

Ten mg/mL α-CHCA in 70:30 ACN: 0.5% TFA (aq) was deposited by spotting 0.5 µL on top of 0.5 μL of the sample for MALDI MS and MS/MS experiments on the MALDI Synapt instrument. For the timsTOF flex instrument, 1 μL of the sample solution was spotted on an AnchorChip™ target plate and dried at room temperature, then 0.5 μL of 1.4 mg/mL α-CHCA in 85:15 ACN: H_2_O, 0.1% TFA (aq) was spotted on top of the sample for MALDI MS/MS experiments and dried. Then the MALDI spots were washed with 1 μL of 1 mM ammonium dihydrogen phosphate (NH_4_H_2_PO_4_).

#### 3.3.3. Data Processing of MALDI MS Data

Six replicate spectra were acquired for each animal blood type investigated (obtained intravenously or from packaged meat). For the purposes of protein detection and identification in each animal blood type, only one spectrum was used to search for protein matches whilst all replicates were used for statistical analyses and discrimination of blood across the three animal species. Mass spectra were viewed in Mass Lynx, (Waters Corporation, Manchester, UK) and Data Analysis 5.3 (Bruker Daltonik, Bremen, Germany). Spectra were then exported to mMass, an open-source multifunctional mass spectrometry software [15,16] upon conversion of the raw spectra into .txt files and only the peaks with S/N of 10 or above were annotated. Mass lists of known matrix (or matrix cluster, adduct) and trypsin autolysis *m*/*z* peaks were generated and used as an exclusion list for peak annotation. For putative protein identifications, candidate blood proteins were selected for in silico digestion; namely: αHb and βHb chains, Erythrocyte Membrane Protein Band 4, Haptoglobin, Ceruloplasmin, Apolipoprotein, Myoglobin, Glycophorin A, Complement C4 and Albumin. UniProt (https://www.uniprot.org/, accessed 12 January 2022) was used to search for protein sequences of interest and imported into mMass using “sequence tool” where the in-silico proteolysis with trypsin and automatic peak assignment were performed. For blood in packaged meat a MASCOT PMF (peptide mass fingerprint) search was preliminary launched selecting “monoisotopic MH^+^” values, peptide tolerance of 15 ppm, two missed cleavages and trypsin as the proteolytic enzyme, chordata or mammalia as taxonomy when in the presence of chicken or bovine and porcine blood, respectively. Subsequently the same strategy used for intravenous blood was applied to blood in packaged meat.

##### MALDI MS/MS Spectral Identification

MS/MS spectra acquired on the MALDI qTOF Synapt HDMS system instrument were opened in MassLynx and then converted in .txt files to be viewed in mMass for smoothing and peak labelling prior to launching an automatic MASCOT MS/MS search using the same parameters as for the MASCOT PMF search. The mass tolerance for the parent ion and the ion fragments was set at 20 ppm and 30 ppm, respectively. MS/MS spectra acquired on the MALDI timsTOF flex instrument were visualised using the Data Analysis 5.3 software. Profile spectra were exported as text files. The files were then viewed in mMass for MASCOT MS/MS searches and spectral annotation.

##### Data Processing: Principal Component Analysis

Prior to statistical analysis, SpecAlign (Oxford, UK), an open access software tool, was used to pre-process all spectra acquired [17], which were imported as .txt files for spectral pre-processing consisting in baseline correction, noise removal, normalization to the total ion count (TIC) and removing negative peaks, prior to generating an average spectrum for spectral alignment using the PAFFT correlation method (maximum shift set to 20). Post Specalign .txt files were transposed into table format and then imported into MarkerView software 1.2 (Applied Biosystems/MDS Sciex, Concorde, ON, Canada). A minimum intensity of 0.1 was selected, with a maximum number of peaks of 20,000 and only monoisotopic peaks were selected for both unsupervised and supervised PCA.

## 4. Conclusions

In silico digestion of blood specific proteins enables the prediction of proteotypic peptides that may be detected (and then experimentally observed) and used, or species discrimination. This was the strategy used by Kennedy et al. [7] in developing a method for the detection and discrimination of blood provenance, distinguishing between human and three animal species. Confirming or refuting the origin of a questioned blood stain as animal, may be crucial finding in an investigation and would assist with reconstructing the dynamics of the bloodshed, as, for example the UK case Regina v. Susan May and the US case, State v. Leuluaialii, Nos. 96-C-08256-9, 97-C-01391-3 suggest. Given the importance of this intelligence, a more objective and quantitative analysis is desirable. Such analysis was involved in this study with the use of statistical analysis to determine discriminatory panels of species-specific blood markers.

The present study integrated principal component analysis (PCA) in the Kennedy et al. approach [7] showing that following proteotypic peptide predictions exclusively reduces the discriminatory accuracy of the method. Interestingly, the findings from this integrated approach also confirmed the hypothesis made by Kennedy et al. [7] that it is possible to distinguish the origin of animal blood whilst still enabling animal species differentiation. In particular, within the system investigated, intravenous blood can be distinguished from blood found in packaged meat. This intelligence could assist in crime reconstruction by ascertaining the reasons why animal blood, and of a particular origin, was found at the scene. In the present study, PCA plots indicated the panel of biomarkers distinguishing (i) intravenous blood from chicken, porcine and bovine sources; (ii) packaged meat blood from chicken, porcine and bovine, as well as distinguishing packaged meat blood from intravenous blood. Some of these markers were also identified through MALDI MS/MS analyses, thus improving robustness of the blood detection and provenance method developed by Kennedy et al. [7]. An additional study revealed that, for packaged meat blood, these discriminatory capabilities are retained, regardless of the supermarket from which the meat was purchased. Finally, the potential to determine the origin (supermarket) of the blood in the packaged meat was assessed. Bovine packaged meat blood was investigated as an example and data revealed that out of the five supermarket retailers used in the study, it is possible to determine provenance from Sainsbury’s. This could be due to this supermarket using a different food processing/supplier than the other four supermarkets. Morrisons’ and Aldi’s spectral profiles of bovine blood in their packaged meat also appear to cluster away from Sainsbury’s and from each other. However, they would be indistinguishable from Asda’s and Tesco’s, respectively (though the analysis of the packaging may reveal additional intelligence on provenance). Altogether this intelligence could assist further in the investigation of violent crimes where animal blood is potentially involved by proving/disproving the defendant’s claim and in reconstruction of the facts under scrutiny.

However, whilst the findings from the present study are original and contribute to potentially new knowledge, some caution is necessary when evaluating their immediate transferability to real life forensic scenarios. Firstly, it would be important to investigate, in a more comprehensive study, reproducibility of the results by performing and analysing multiple protein digests from (i) the same blood source, especially from blood in meat packets, (ii) blood from multiple packets of the same cut as well as from different animal parts and (iii) blood from both fresh meat and meat that had been frozen. Additionally, it is important to bear in mind that it is not yet possible to assimilate definitively the intravenous blood collected from the animal jugular to the blood in a scenario in which the animal is shot at a scene, although jugular blood is the closest approximation in this paper until such studies can be performed. It would also be important to investigate the MALDI spectra profiles of blood originating from animals shot in different body locations, and with a different tissue/muscular tissue depth, to definitely establish differentiation from the spectral profiles of blood from packaged meat.

## Figures and Tables

**Table 1:** Bovine intravenous blood peptides putatively identified with a mass accuracy ranging between −3.7 and 4.7 ppm.

Experimental *m*/*z*	Theoretical *m*/*z*	Putative Peptide Match	Mass Accuracy (ppm)	Proteotypic	Protein
639.394	639.394	VKAHGK	0	NO	βHb
767.487	767.489	VKAHGKK	2.6	NO	βHb
950.506	950.509	AAVTAFWGK	3.2	YES	βHb
1071.554	1071.554	MFLSFPTTK	0	NO	αHb
1101.627	1101.629	VLSAADKGNVK	−1.8	YES	αHb
1225.626	1225.625	KVLDSFSNGMK	−0.8	YES	βHb
1274.724	1274.726	LLVVYPWTQR	−1.6	NO	βHb
1328.715	1328.717	VKVDEVGGEALGR	1.5	YES	βHb
1477.795	1477.802	VVAGVANALAHRYH	4.7	NO	βHb
1529.733	1529.734	VGGHAAEYGAEALER	0.7	NO	αHb
1752.900	1752.899	MLTAEEKAAVTAFWGK	−0.6	YES	βHb
1833.890	1833.891	TYFPHFDLSHGSAQVK	0.5	NO	αHb
1868.961	1868.954	NFGKEFTPVLQADFQK	−3.7	YES	βHb
2089.953	2089.953	FFESFGDLSTADAVMNNPK	0	YES	βHb
2284.129	2284.126	TYFPHFDLSHGSAQVKGHGAK	−1.3	YES	αHb

**Table 2:** Putatively identified peptides from the MALDI MS spectra of proteolysed porcine intravenous blood.

Experimental *m*/*z*	Theoretical *m*/*z*	Mass Accuracy (ppm)	Peptide Sequence	Proteotypic	Protein
767.487	767.487	0	VKAHGKK	NO	βHb
1041.542	1041.544	1.9	MFLGFPTTK	YES	αHb
1115. 643	1115.642	−0.9	VLSAADKANVK	YES	αHb
1238.680	1238.685	4.0	AHGQKVADALTK	YES	αHb
1243.679	1243.679	0	YELDKAFSDR	YES	αHb
1265.826	1265.830	3.2	LLGNVIVVVLAR	NO	αHb
1274.724	1274.726	1.6	LLVVYPWTQR	NO	βHb
1314.670	1314.665	−3.8	VNVDEVGGEALGR	NO	βHb
1422.700	1422.708	5.6	VGGQAGAHGAEALER	YES	αHb
1449.784	1449.796	8.3	VVAGVANALAHKYH	NO	βHb
1628.908	1628.912	2.5	VLSAADKANVKAAWGK	YES	αHb
1813.977	1813.981	2.2	VLQSFSDGLKHLDNLK	YES	βHb
1876.897	1876.898	0.5	TYFPHFNLSHGSDQVK	YES	βHb
1935.967	1935.978	5.7	AAWGKVGGQAGAHGAEALER	YES	βHb
2045.920	2045.927	3.4	FFESFGDLSNADAVMGNPK	YES	βHb
2237.156	2237.167	4.9	AVGHLDDLPGALSALSDLHAHK	YES	βHb
2318.244	2318.250	2.6	VLQSFSDGLKHLDNLKGTFA K	YES	βHb
2398.167	2398.167	0	TYFPHFNLSHGSDQVKAHGQK	YES	αHb
2445.216	2445.234	7.4	VGGQAGAHGAEALERMFLGFPTTK	YES	αHb

**Table 3:** Putatively identified peptides from the MALDI MS spectra of proteolysed chicken intravenous blood. All protein matches found in the intravenous chicken blood were proteotypic to this species.

Experimental *m*/*z*	Theoretical *m*/*z*	Putative Peptide Match	Mass Accuracy (ppm)	Proteotypic	Protein
920.489	920.495	LSDLHAHK	6.5	YES	αHb
1036.560	1036.567	VLTSFGDAVK	6.8	YES	βHb
1085.533	1085.534	MFTTYPPTK	0.9	YES	βHb
1288.728	1288.741	LLIVYPWTQR	10.1	YES	βHb
1302.635	1302.647	VNVAECGAEALAR	9.2	YES	βHb
1645.778	1645.782	IAGHAEEYGAETLER	2.4	YES	αHb
1704.959	1704.964	VLSAADKNNVKGIFTK	2.9	YES	αHb
2121.124	2121.155	VVAALIEAANHIDDIAGTLSK	14.6	YES	αHb
2226.142	2226.138	FFASFGNLSSPTAILGNPMVR	−1.8	YES	βHb

**Table 4:** Animal species discriminatory supervised PCA ion signals (from Figure 4) selected putative identification and MS/MS confirmation for blood in packaged meat. (NI: Not Identified; NA: not applicable).

Species	Experimental *m*/*z* (Th)	Putative ID and UniProt Accession No.	Mass Accuracy (ppm)	MS/MS ID (and Accession No.)	Peptide Sequence
Bovine	1198.718	Actin (P62739)	−10.4	Actin (P62739)	AVFPSIVGRPR
Bovine	1348.768	Carbonic anhydrase 3 (Q3SZX4)	−6.2	Carbonic anhydrase 3 (Q3SZX4)	NWRPPQPIKGR
Bovine	1669.851	Myoglobin (P02192)	−8.6	Myoglobin (P02192)	ALELFRNDMAAQYK
Bovine	1771.932	NI	−4.3	Carbonic anhydrase 3 (Q3SZX4)	TLYSSAENEPPVPLVR
Bovine	1790.916	Actin (P62739)	−13.5	Actin (P62739)	SYELPDGQVITIGNER
Bovine	2280.178	Myoglobin (P02192)	−8.2	Myoglobin (P02192)	ALELFRNDMAAQYKVLGFHG
Porcine	758.576	NI		(Lipid)	NA
Porcine	796.533	NI		(Lipid)	NA
Porcine	1536.823	NI		NI	NA
Porcine	1541.766	Beta-Enolase (Q1KYT0)	−1.2	Beta-Enolase (Q1KYT0)	LAQSNGWGVMVSHR
Porcine	2458.314	NI		NI	NA
Porcine	2463.248	NI	−0.3	Beta-Enolase (Q1KYT0)	AAVPSGASTGIYEALELRDG DKSR
Porcine	2123.125	NI		Fructose-bisphosphate aldolase (Q6UV40)	IGEHTPSSLAIMENANVLAR
Chicken	1314.710	NI		NI	NA
Chicken	1749.799	GAPDH (P00356)	−7.0	GAPDH (P00356)	LVSWYDNEFGYSNR
Chicken	1936.042	NI		NI	NA

## Data Availability

Not applicable.

## Supplementary Materials

The following supporting information can be downloaded at: https://www.mdpi.com/article/10.3390/molecules27072039/s1, Figure S1: Annotated MALDI qTOF Synapt HDMS MS/MS spectra of intravenous animal blood markers at nominal *m*/*z* 1329 (**A**-bovine blood), 1423 (**B**-porcine blood) and 1646 (**C**-chicken blood). Table S1: List of *m*/*z* ions submitted to MS/MS analysis selected from the supervised PCA analysis. Table S2: MALDI MS/MS spectral identifications of discriminatory ion signals selected from the PCA analysis in Figure 4, for packaged meat blood from different animal species.

## Sample Availability

Samples of the compounds are not available.
