# Supplementary material for: Forensic Discrimination of Differentially Sourced Animal Blood Using a Bottom-Up Proteomics Based MALDI MS Approach
Source: Molecules. 2022 Mar 22;27(7):2039. doi: 10.3390/molecules27072039 (PMC9000818; doi:10.3390/molecules27072039)
Supplement: Supplementary file 1 [file molecules-27-02039-s001.zip › molecules-1586249-supplementary.pdf]

Supplementary Information

Table S1. List of *m/z* ions submitted to MS/MS analysis selected from the supervised PCA analysis. Identification/confirmation identity of intravenous chicken, bovine, and porcine blood Hb biomarkers is reported.

Species	Ion Signal *m/z* (Th)	Protein and Peptide Sequence	Ion fragments detected
Bovine	1328.727	βHb VKVDEVGGEALGR	y4; y7-y9; b5; b10-b12
Chicken	1645.791	αHb IAGHAEEYGAETLER	b5-7; b9-10; b12-14; y3-7; y7-9; y11; y13
Porcine	1422.714	αHb VGGQAGAHGAEALER	b4-b14; y3-y14
Bovine	1529.745	αHb VGGHAAEYGAEALER	y1; y4; y7-y8; y11-y12; b4-b14
Porcine	1274.734	βHb LLVVYPWTQR	y2-y3, y5-y7, b4-b5, b7-b9

Table S2. MALDI MS/MS spectral identifications of discriminatory ion signals selected from the PCA analysis in Figure 4, for packaged meat blood from different animal species.

Species	Ion signal *m/z* (Th)	Protein and Peptide Sequence	Ion fragments detected
Bovine	1198.718	Actin AVFPSIVGRPR	b2-b10; y1-y3; y5-y9
Bovine	1790.916	Actin AVFPSIVGRPR	b2-b5; b7-b13; y1-y2; y5-y14;
Bovine	1348.768	Carbonic anhydrase 3 NWRPPQPIKGR	b2-b3; b6; b8-b10, y1-y3; y5; y7-y10
Bovine	1771.932	Carbonic anhydrase 3 NWRPPQPIKGR	b2--b12; b14-b15; y1-y4; y7-y16; y15
Bovine	1669.851	Myoglobin ALELFRNDMAAQYK	b2-b13; y2-y13
Bovine	2280.178	Myoglobin ALELFRNDMAAQYK	b2-b13; b15-b19; y3; y5-y13; y16-y17
Porcine	1541.766	Beta-Enolase LAQSNGWGVMVSHR	a9,a13; y1-y4,y8,y10; b8-b13
Porcine	2123.125	Fructose-bisphosphate aldolase IGEHTPSSLAIMENANVLAR	b4-b6; b8-b19; y4-y8; y9-y18
Porcine	2463.248	Beta-Enolase AAVPSGASTGIYEALELRDG DKSR	y1,y5,y7-y8,y11,y15; b18-b19,b21,b23; c18-c19
Chicken	1749.799	GAPDH LVSWYDNEFGYSNR	a11;y1-y12;b3,b5-b11

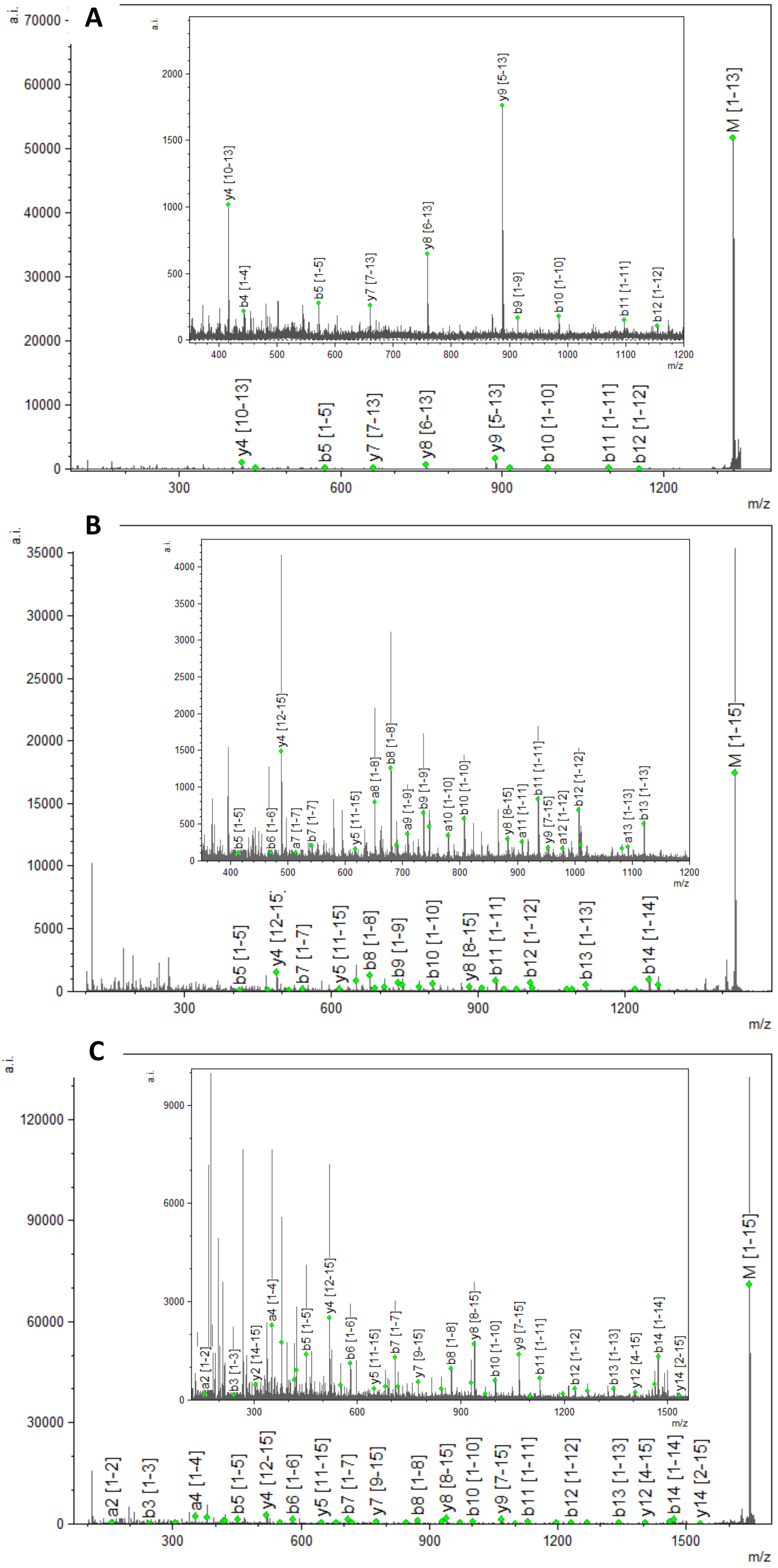

A
B
C
a.i.
m/z
M [1-13]
y4 [10-13]
b4 [1-4]
b5 [1-5]
y7 [7-13]
y8 [6-13]
y9 [5-13]
b9 [1-9]
b10 [1-10]
b11 [1-11]
b12 [1-12]
M [1-15]
y4 [12-15]
b6 [1-6]
a7 [1-7]
b7 [1-7]
y5 [11-15]
a8 [1-8]
b8 [1-8]
a9 [1-9]
b9 [1-9]
a10 [1-10]
y8 [8-15]
a11 [1-11]
y9 [7-15]
a12 [1-12]
a13 [1-13]
b13 [1-13]
b14 [1-14]
a2 [1-2]
b3 [1-3]
y2 [14-15]
a4 [1-4]
y7 [9-15]
y12 [4-15]
y14 [2-15]

Figure S1. Annotated MALDI qTOF Synapt HDMS MS/MS spectra of intravenous animal blood markers at nominal *m/z* 1329 (**A**-bovine blood), 1423 (**B**-porcine blood) and 1646 (**C**-chicken blood). Annotations for b and y ions are reported and zoom in insets are shown for each MS/MS spectrum.
